# Osteogenic Potential of Monosodium Urate Crystals in Synovial Mesenchymal Stem Cells

**DOI:** 10.3390/medicina58121724

**Published:** 2022-11-24

**Authors:** Karina Martínez-Flores, Ricardo Plata-Rodríguez, Anell Olivos-Meza, Ambar López-Macay, Javier Fernández-Torres, Carlos Landa-Solís, Yessica Zamudio-Cuevas

**Affiliations:** 1 Laboratorio de Líquido Sinovial, Instituto Nacional de Rehabilitación Luis Guillermo Ibarra Ibarra, Mexico City 14389, Mexico; 2 Facultad de Química, UNAM, Circuito Exterior S/N, Coyoacán, Cd. Universitaria, Mexico City 04510, Mexico; 3 Servicio de Ortopedia del Deporte y Artroscopía, Instituto Nacional de Rehabilitación Luis Guillermo Ibarra Ibarra, Mexico City 14389, Mexico; 4 Unidad de Ingeniería de Tejidos, Terapia Celular y Medicina Regenerativa, Instituto Nacional de Rehabilitación Luis Guillermo Ibarra Ibarra, Mexico City 14389, Mexico

**Keywords:** monosodium urate crystals, gout, osteodifferentiation, synovial membrane, mesenchymal stem cells

## Abstract

*Background and Objectives:* Deposits of monosodium urate (MSU) crystals due to increased levels of uric acid (UA) have been associated with bone formation and erosion, mainly in patients with chronic gout. The synovial membrane (SM) comprises several types of cells, including mesenchymal stem cells (SM-MSCs); however, it is unknown whether UA and MSU induce osteogenesis through SM-MSCs. *Materials and Methods:* Cultures of SM were immunotyped with CD44, CD69, CD90, CD166, CD105, CD34, and CD45 to identify MSCs. CD90+ cells were isolated by immunomagnetic separation (MACS), colony-forming units (CFU) were identified, and the cells were exposed to UA (3, 6.8, and 9 mg/dL) and MSU crystals (1, 5, and 10 μg/mL) for 3 weeks, and cellular morphological changes were evaluated. IL-1β and IL-6 were determined by ELISA, mineralization was assessed by alizarin red, and the expression of Runx2 was assessed by Western blot. *Results:* Cells derived from SM and after immunomagnetic separation were positive for CD90 (53 ± 8%) and CD105 (52 ± 18%) antigens, with 53 ± 5 CFU identified. Long-term exposure to SM-MSCs by UA and MSU crystals did not cause morphological damage or affect cell viability, nor were indicators of inflammation detected. Mineralization was observed at doses of 6.8 mg/dL UA and 5 μg/mL MSU crystals; however, the differences were not significant with respect to the control. The highest dose of MSU crystals (10 μg/mL) induced significant Runx2 expression with respect to the control (1.4 times greater) and SM-MSCs cultured in the osteogenic medium. *Conclusions:* MSU crystals may modulate osteogenic differentiation of SM-MSCs through an increase in Runx2.

## 1. Introduction

Joint damage is a characteristic of erosive arthropathies, such as tophaceous gout, and is associated with musculoskeletal disability. Joint deformity and disability progress as more joints are affected and the tophi increase in size and number [1,2]. The association between monosodium urate (MSU) crystal deposition and joint damage in patients with chronic gout has been determined by the presence of tophi adjacent to sites of cartilage loss and joint destruction. Tophaceous deposits can erode bones, cartilage, and tendons, causing significant structural damage [3].

During an acute gout attack, proinflammatory cytokines, such as IL-1β, are involved in the promotion of bone damage and in the activation and differentiation of osteoclasts, and induce the production of enzymes that degrade the extracellular matrix (ECM), including metalloproteinases, because of chronic inflammation [4].

The stimulation of osteoclast precursors by MSU crystals does not directly promote the formation of osteoclasts; this process can occur indirectly through the stromal cells of tophi. MSU crystals inhibit the gene and protein expression of osteoprotegerin (OPG) or osteoclastogenesis inhibitor factor in stromal cells and fibroblast-like synoviocytes (FLSs) without altering the gene expression of the receptor activator ligand for nuclear factor kappa-B (RANKL); thus, an imbalance in RANKL–OPG may lead to osteoclast development from precursors [5].

On the other hand, MSU crystals in osteoblasts promote the expression of RANKL–OPG, inducing osteoclastogenesis and bone resorption, and there is less osteoblast differentiation, since human osteoblasts stimulated by MSU crystals and IL-1β decrease osteocalcin formation and alkaline phosphatase activity [6].

MSU crystals contribute to bone erosion in gout through the formation and activation of osteoclasts and decreased viability, function, and differentiation of osteoblasts, due to a reduction in mineralization and the expression of genes related to osteoblast differentiation, such as Runx2, Osterix (Sp7), Bone Sialoprotein (Ibsp), and Osteocalcin (Bglap). This suggests that bone erosion in gout occurs at the tophus–bone interface through alterations in the physiological turnover of bone, with excessive new bone formation, and activation of osteoclasts, leading to reduced osteoblast differentiation [7].

Currently, there are no studies on mesenchymal stem cells (MSCs) from the synovial membrane (SM) evaluating the impact of uric acid (UA) and MSU crystals on osteodifferentiation. Some reports indicate that hyperuricemia induces the differentiation of placenta-derived MSCs in neuronal cells in vitro [8], and other studies have reported the effect of UA on the osteogenic and adipogenic differentiation of human MSCs isolated from bone marrow (hBMSCs) through the 11β-hydroxysteroid dehydrogenase type 1 (11β-HSD1), alpha-1 subunit of nucleus binding factor, Cbfa1, Runx2, and Wnt signaling pathways [9,10].

To date, most gout models have been used to study inflammatory processes in order to clarify the pathogenic and molecular mechanisms of MSU crystals in cells that comprise the SM, such as FLSs. The participation of MSCs in this process has been poorly studied, especially in osteodifferentiation induced by UA exposure or MSU crystal deposition. There is little information about the mechanisms of osteogenesis induced by UA and MSU crystals in gout in terms of modulating the expression of proteins that favor the bone erosion and new bone formation in affected joints. Understanding the molecular mechanisms of MSCs in terms of amplifying their response to soluble UA (sUA) and crystals will help in better defining their role in the regulation of osteogenesis in chronic gout. Therefore, we hypothesized that sUA and/or MSU crystals play a role in the regulation of osteodifferentiation during the chronic phase of the disease.

## 2. Materials and Methods

A comparative, analytical, cross-sectional, and non-probabilistic experimental study was carried out [11].

Samples were obtained from 6 patients (*n* = 6) with anterior cruciate ligament tear confirmed by diagnostic anterior drawer and Lachman tests [12] by physicians at the Arthroscopy Service of the Instituto Nacional de Rehabilitación. At the time of recruitment, all the subjects gave informed consent for inclusion before participating in the study. Patients who underwent arthroscopic surgery donated their remaining SM tissue. The inclusion criteria for patients were as follows: a body mass index (BMI) lower than 25 kg/m^2^, no existing gout condition or diagnosed disease, and a serum UA level lower than 416.36 μmol/L (7 mg/dL). Additionally, individuals with type 2 diabetes, chronic renal failure, or any other rheumatic disease were not included in the study. The study was conducted in accordance with the Declaration of Helsinki, and the protocol was approved by the Ethics Committee CONBIOETICA-09-CEI-031-20171207 (Project identification 23/21) of the INR-LGII, on 12 July 2021.

### 2.1. Cell Isolation, Culture, and Immunophenotyping

The collected synovial tissue was washed with 1× PBS supplemented with 10% penicillin/streptomycin (PS), followed by mechanical disintegration with a scalpel in 60 × 15 mm Petri dishes. Seeding by explant was performed, in which the SM fragments were added to DMEM-F12 culture medium (Gibco, Life Technologies, Carlsbad, CA, USA) supplemented with 20% fetal bovine serum (FBS) and 1% PS. The explants were incubated at 37 °C in an atmosphere of 95% humidity and 5% CO_2_. At 24 h, nonadherent cells were removed by replacing the medium with fresh culture medium; this process was conducted every third day until the cells were 80–90% confluent. Subcultures were performed, and in the second passage, the cells were immunophenotyped using flow cytometry (BD FACSCalibur) with anti-CD90 antibodies coupled to fluorescein isothiocyanate (FITC; BD Pharmingen 561988, San Diego, CA, USA), CD105 coupled to phycoerythrin (PE; BD Pharmingen 560839), CD73 coupled to peridinin chlorophyll (PerCP-C; BD Pharmingen 581280), CD117 allophycocyanin (APC; BD Pharmingen 341106), CD14–FITC (Thermo Scientific 1-82074, Waltham, MA, USA), CD34–PE (BD Pharmingen 555822), CD45–FITC, (BD Pharmingen 555482), CD166–PE (BD Pharmingen 559263), CD271–FITC (Miltenyi Biotec 130098, Bergisch Gladbach, Germany), and CD31–FITC (Thermo Scientific 1-80360).

### 2.2. Immunomagnetic Separation and Immunophenotyping Assay

From 2 × 10^6^ cells in the third passage, the CD90+ subpopulation was isolated by MidiMACS immunomagnetic separation (Miltenyi Biotec, Bergisch Gladbach, Germany) using anti-CD90 (Miltenyi Biotec) coupled to magnetic beads (MACS MicroBeads, Miltenyi Biotec) and separation columns (MS Columns, Miltenyi Biotec). Cells were incubated with anti-CD90 conjugated to 50 nm superparamagnetic particles for 15 min at 4 °C protected from light. Then, the cells were passed through magnetic separation columns and recovered by negative selection (CD90- cells); the column was subsequently removed from the magnetic field to recover CD90+ cells by elution. CD90+ cells were expanded and used for osteodifferentiation assays.

### 2.3. Characterization of CD90+ Cells and Colony-Forming Unit (CFU) Assay

CD90+ cells were cultured until confluent and were immunophenotyped using flow cytometry (BD FACSCalibur). The cells were phenotyped with CD90–FITC, CD105–PE, CD73–PerCP-Cy, and CD117–APC; other cells were labeled with CD14–FITC and CD34–PE, CD45–FITC and CD166–PE, and CD271–FITC and CD31–FITC. To identify whether the SM-MSCs had the capacity to form colonies, 1000 cells of the CD90+ subpopulation were cultured in 6-well plates for 14 days [13] in DMEM-F12 supplemented with 20% FBS and 1% PS; the cells were incubated at 37 °C in an atmosphere with 95% humidity and 5% CO_2_. For the identification of CFUs, the crystal violet dye (Sigma-Aldrich C3886-25G, St. Louis, MO, USA) incorporation technique was used [14]. CFUs were identified and quantified under a SteReo Discovery V.20 Zeiss stereoscopic microscope.

### 2.4. Cell Stimulation

CD90+ cells were exposed to UA (Sigma-Aldrich U2625, St. Louis, MO, USA) at concentrations of 3, 6.8, and 9 mg/dL, simulating states of hypouricemia, normouricemia, and hyperuricemia [15], respectively. The MSU crystals were synthetized, characterized, and sterilized according to [16]. The absence of microbial contaminants was confirmed by negative cultures for microorganisms and endotoxins. Cells were exposed to MSU crystals in concentrations of 1, 5, and 10 μg/mL in DMEM-F12 medium supplemented with 20% FBS and 1% PS. Cells stimulated with osteogenic culture medium (StemMACS OsteoDiff Media, human, Miltenyi Biotec, Friedrich-Ebert-Strasse, Bergish Gladbach, Germany) were used as positive control. All experimental groups were compared with a control group (cells without stimuli). All culture media were changed every third day for 3 weeks.

### 2.5. Analysis of Cell Viability

After stimulating the cells with the established concentrations of UA, MSU crystals, and osteogenic induction medium for the established time, the supernatants were removed from the culture dishes and stored at −20 °C for further studies. The cells were washed with PBS and fixed with 2.5% glutaraldehyde (ICN Biomedicals, Inc., Chillicothe Rd., Aurora, OH, USA) for 10 min. Then, the cells were washed again and stained with crystal violet. The dye was quantified through dissolution with acetic acid glacial (Fermont 03011 Productos Químicos, Monterrey, S. A de C.V, Mexico) [13]. The absorbance was measured with a microplate reader at 595 nm (iMark^TM^, Bio-Rad, serial no. 10176, Hercules, CA, USA). Absorbance readings were normalized with respect to the control, converting the values into 100% viable cells [17].

### 2.6. Determination of Calcium Nodules

To determine the mineralization of the ECM of SM-MSCs exposed to the established conditions, staining with alizarin red (Sigma-Aldrich A5533, St. Louis, MO, USA) was performed [18]. The formation of mineralized ECM was identified by intense red regions corresponding to Ca^2+^ nodules in pleomorphic clusters [19] under an Evos microscope (L1113-178C-173, Life Technologies Corp., Bothell, WA, USA). The cells were washed with PBS and fixed with 4% paraformaldehyde. Then, the cells were washed, allowed to dry, and stained with 2% alizarin red (Sigma-Aldrich A5533-25G, St. Louis, MO, USA) for 20 min in a shaker (Compact Rocker, Bio-Rad Labnet International Inc., Woodbridge, NY, USA). Subsequently, alizarin red staining was quantified by removing the dye with isopropanol for 20 min under orbital agitation, and the absorbance of the dye was measured using a microplate reader (iMark^TM^, Bio-Rad, serial no. 10176, Tokyo, Japan) at 415 nm; the absorbance of the positive control was used as a reference, based on the red regions, which were quantified and used as a reference for 100% calcium nodules.

### 2.7. Analysis of Proinflammatory Cytokines

IL-1β and IL-6 were quantified by ELISA using the supernatants. The commercial Human IL-1β Standard ABTS ELISA Development Kit (Peprotech, 900-K95, Cedarbrook Drive, Cranbury, NJ, USA) was used, and IL-6 was quantified using the Human IL-6 Standard ABTS ELISA Development Kit (900-K16, Cedarbrook Drive, Cranbury, NJ, USA). Avidin peroxidase, 2,2′-azino-bis (3-ethylbenzothiazoline-6-sulfonic acid) ABTS (Sigma-Aldrich A9941, St. Louis, MO, USA) was used as a chromogenic substrate. The samples were read at 405 nm using a microplate reader (iMark^TM^, Bio-Rad, serial no. 10176, Tokyo, Japan). The results were compared to the standard curve for IL-1β and IL-6 and expressed in pg/mL.

### 2.8. Extraction and Quantification of Total Protein

The cells were washed with PBS (4–8 °C) and then mechanically lysed. The lysate was centrifuged at 23,000× *g* for 15 min at 4 °C, and the cell pellet was resuspended in lysis buffer M-PER Mammalian protein extraction reagent (Thermo Fisher Scientific, Pierce Biotechnology, Rockford, IL, USA) containing 1 M DTT, 1 M PMSF, protease inhibitor (Complete, Roche Diagnostics GmbH, Mannheim, Germany), and phosphatase inhibitor (PhosSTOP, Roche Diagnostics GmbH, Mannheim, Germany). Cells were sonicated (Tmishion, 008 China, Rue de la Caille, Nuaillé, France) for 15 min and subsequently centrifuged at 23,000× *g* for 10 min at 4 °C. The supernatant containing the total protein was aliquoted and stored at −80 °C. The protein concentration was determined using Quick Start Bradford 1× dye reagent (#5000205 Bio-Rad Laboratories, Inc., Hercules, CA, USA). A bovine serum albumin (BSA) (P6154-Biowest, Nuaillé-France) standard curve from 25 to 2000 µg/mL was used. The proteins were incubated for 2 h at 37 °C. Absorbance was measured using a microplate reader (iMark^TM^, Bio-Rad, 10176, Tokyo, Japan) at 595 nm.

### 2.9. Analysis of Runx2 Protein Expression by Western Blot

Separation of 20 µg total protein was carried out by electrophoresis (120 V for 120 min) on 10% polyacrylamide gels containing 0.1% SDS. Subsequently, the protein was transferred to nitrocellulose membranes for 10 min at 25 V and 1 ampere in a Trans-Blot Turbo Transfer System (690BR4448 Bio-Rad Laboratories, Inc., Hercules, CA, USA). The membranes were blocked for 1 h under agitation in TBS-Tween containing 5% BSA. Subsequently, the membranes were incubated overnight under agitation at 4 °C with anti-Runx2 (mouse, monoclonal, Abcam 76956, Boston, MA, USA) at a 1:1000 dilution and β-actin-peroxidase (mouse, monoclonal, A3854; Sigma-Aldrich, St. Louis, MO, USA) at a 1:10,000 dilution. A secondary antibody coupled to anti-mouse IgG peroxidase (Abcam 97200) was used at a 1:10,000 dilution. Immunodetection was performed using Immobilon Western Chemiluminescent HRP Substrate (Millipore Corp., Billerica, MA, USA). The blots were visualized by exposure to CL-XPOSURE radiographic plates (Pierce Chemical, Rockford, IL, USA) and scanned with VueScan software (HP DeskJet 2135, Korea). Densitometric analysis was performed with ImageJ version 1.53e (National Institutes of Health, Bethesda, MD, USA). β-actin was used as a load control.

### 2.10. Statistical Analysis

Each experiment was performed independently at least 3 times using cells from different patients (*n* = 6). Statistical analysis of the results was performed with GraphPad Prism 9.1.2. One-way analysis of variance (ANOVA) was followed by Dunnett’s or Tukey’s post hoc test. A *p* < 0.05 was considered statistically significant.

## 3. Results

Among the patients, 66% were male and 34% were female. The average age of the participants was 37 ± 13 years. None of them had a history of gout or hyperuricemia, none had clinical evidence of gout, and their synovial fluid did not indicate inflammation at the time of arthroscopy surgery nor contained MSU crystals. SM-derived cells presented a fibroblastic, fusiform phenotype with cytoplasmic extensions. Growth in groups was observed; a heterogeneous population of cells with oligo- and polydendritic morphology was also identified. Microscopic observation showed that fibroblasts became the predominant cells in culture after 10–15 days (Supplementary Figure S1).

### 3.1. Immunophenotypic Characterization of Cells Isolated from SM

**Figure 1:**
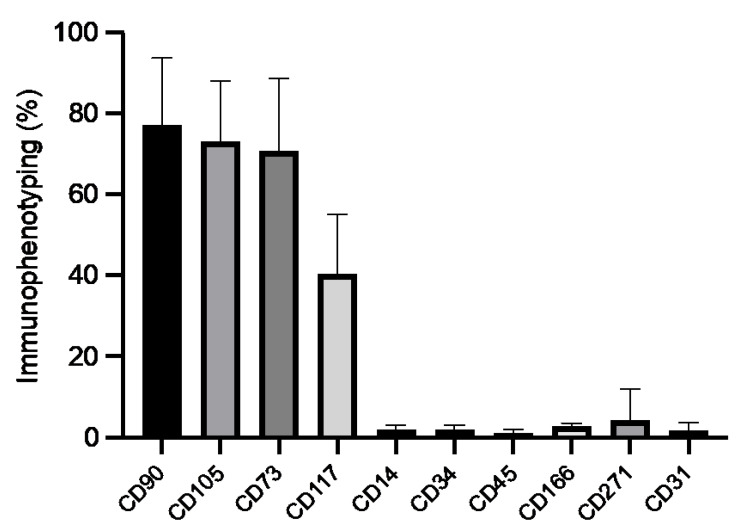
Immunophenotyping of SM-derived cells. SM-derived cells showed abundant CD90, CD105, and CD73 (mesenchymal) expression and lower CD117, CD34, CD45 (hematopoietic), CD271 (neural), and CD31 (endothelial) expression. Values represent the mean ± standard deviation of independent experiments (*n* = 6).

Among the isolated cells, 77 ± 17% expressed CD90, 73 ± 15% expressed CD105, 71 ± 18% expressed CD73, and 40 ± 15% expressed CD117. To a lesser extent, 2 ± 1% expressed CD14, 2 ± 1% expressed CD34, 1 ± 1% expressed CD45, 3 ± 1% expressed CD166, 4 ± 8% expressed CD271, and 2 ± 2% expressed CD31 (Supplementary Figure S2). More than 70% of cells expressed the cellular markers CD90, CD105, and CD73; less than 50% expressed CD117; and less than 5% expressed CD14, CD34, CD45, CD166, CD271, and CD31 (Figure 1).

### 3.2. Immunomagnetic Separation of CD90+ Cells

Cells isolated from the SM and separated by an immunomagnetic column were shown to be mostly CD90+ cells. A total of 1,860,000 ± 922,395 CD90+ cells were isolated from each synovial culture from different patients. Flow cytometry showed that cells derived from the SM after immunomagnetic separation were positive for CD90 (53 ± 8%) and CD105 (52 ± 18%) antigens, among which 53 ± 5 CFUs were identified in multiple clusters (Supplementary Figure S3).

### 3.3. Effect of UA and MSU Crystals on the Viability of SM-MSCs

**Figure 2:**
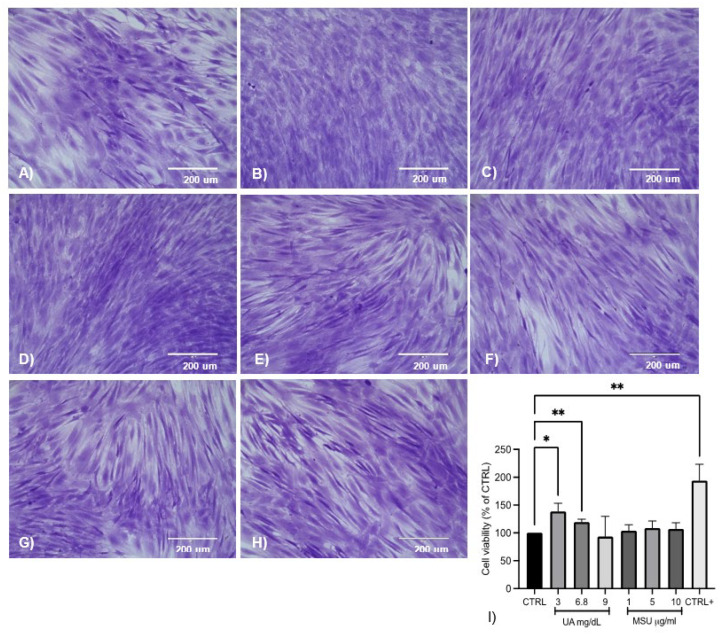
Morphology of SM-MSCs at different concentrations of UA and MSU crystals. Bright-field microscopy of SM-MSCs cultured with UA and MSU crystals. (**A**) Control, (**B**) 3 mg/dL UA, (**C**) 6.8 mg/dL UA, (**D**) 9 mg/dL UA, (**E**) 1 µg/mL MSU crystals, (**F**) 5 µg/mL MSU, (**G**) 10 µg/mL MSU, and (**H**) osteogenic medium (20×). Representative images of independent experiments (*n* = 6). (**I**) Cell viability increased in cultures treated with 3 and 6.8 mg/dL UA and in cells cultured in osteogenic medium. The data represent the mean ± SD of independent experiments (* *p <* 0.05 vs. control; ** *p <* 0.001 vs. control).

Different stimuli did not affect the morphology of the cells. The number of cells increased significantly, by 38 and 19%, in media supplemented with UA at concentrations of 3 and 6.8 mg/dL, respectively, compared to the control. The cell population decreased by 7% with respect to the control using 9 mg/dL UA. MSU crystals at a dose of 5 µg/mL increased the cell population by 8%. Likewise, the cells exposed to 1 and 10 µg/mL MSU crystals exhibited a tendency toward increased viability (4 and 7%, respectively); however, these changes were not significant with respect to the control. The cells exposed to the osteogenic medium showed a significant increase in cell viability (94%) with respect to the control (Figure 2).

### 3.4. Effect of UA and MSU Crystals on the Formation of Calcium Nodules

**Figure 3:**
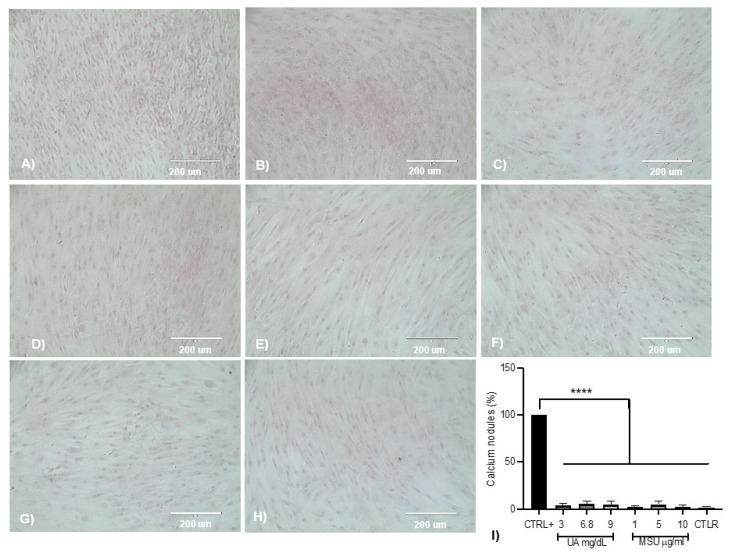
Effect of UA and MSU crystals on the formation of calcium nodules. (**A**) Osteogenic induction control, (**B**) 3 mg/dL UA, (**C**) 6.8 mg/dL UA, (**D**) 9 mg/dL UA, (**E**) 1 µg/mL MSU crystals, (**F**) 5 µg/mL MSU crystals, (**G**) 10 µg/mL MSU, and (**H**) control. Bright-field microscopy; cell culture with red-stained calcium nodules (20×), representative of independent experiments (*n* = 6). The formation of calcium nodules was observed to be high in the positive control (osteogenic medium); in the cells cultured in media containing UA and MSU crystals, the effect was null, as was that in the control. (**I**) Alizarin red staining was quantified by removing the dye with isopropanol. The data represent the mean ± SD of independent experiments (**** *p* < 0.00001 vs. control +).

Few mineralized nodules were observed in the control with respect to cells stimulated with osteogenic medium; in those cells stimulated with 3 mg/dL UA, greater mineralization was observed than in the control and was similar to the positive control. In cells stimulated with 6.8 and 9 mg/dL, limited mineralization was observed with respect to the positive control based on the intensity of alizarin red dye. In the cells exposed to MSU crystals (1, 5, and 10 µg/mL), lower-intensity mineralization was observed, as determined by the scarce red coloration, a finding that was observed in the control. Experiments with UA at concentrations of 3, 6.8, and 9 mg/dL showed less calcium nodule formation compared to the positive control. Likewise, in the cells exposed to MSU crystals, less nodule formation was observed with respect to the positive control: 1 µg/mL MSU crystals generated 2% mineralization, 5 µg/mL MSU crystals generated 5%, and 10 µg/mL generated 3% mineralization. In the control cells, there was a smaller percentage of calcium nodules (Figure 3).

### 3.5. Effect of UA and MSU Crystals on Inflammation in SM-MSCs

**Figure 4:**
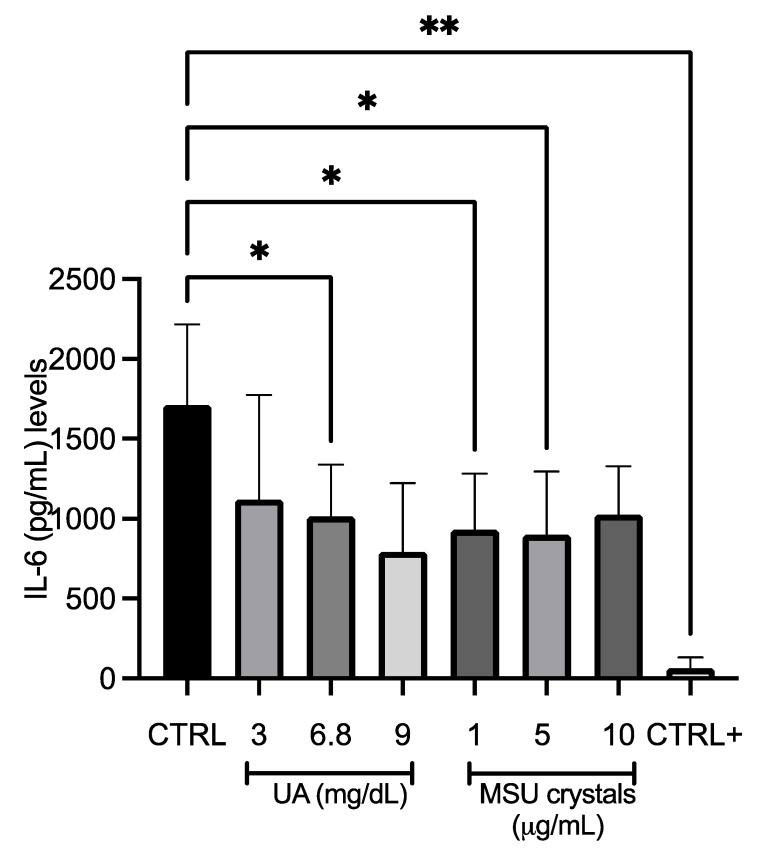
Effect of UA and MSU crystals on IL-6 production by SM-MSCs. Quantification of IL-6 in SM-MSCs treated with different stimuli, i.e., UA (3, 6.8, and 9 mg/dL), MSU crystals (1, 5, and 10 µg/mL), osteogenic medium (control +), and control. The data represent the mean ± SD of independent experiments (*n* = 6) (* *p* < 0.05 vs. control; ** *p* < 0.001 vs. control).

IL-1β levels were not detected at any UA and MSU crystal concentrations used or in cells exposed to osteogenic differentiation medium (data not shown). For IL-6, in cells exposed to 3 mg/dL UA, the concentration was 1119 ± 656 pg/mL; in cells exposed to 6.8 mg/dL UA, the concentration was 1013 ± 327 pg/mL; and in cells stimulated with 9 mg/dL UA, the concentration was 793 ± 430 pg/mL—all of which were lower than compared to control cells (1712 ± 503 pg/mL). In SM-MSCs stimulated with 1, 5, and 10 µg/mL MSU crystals, the IL-6 levels were 930 ± 352, 899 ± 397, and 1026 ± 302 pg/mL, respectively; in cells exposed to osteogenic differentiation medium, the IL-6 concentration was 63 ± 68 pg/mL (Figure 4).

### 3.6. Analysis of Runx2 Protein Expression

**Figure 5:**
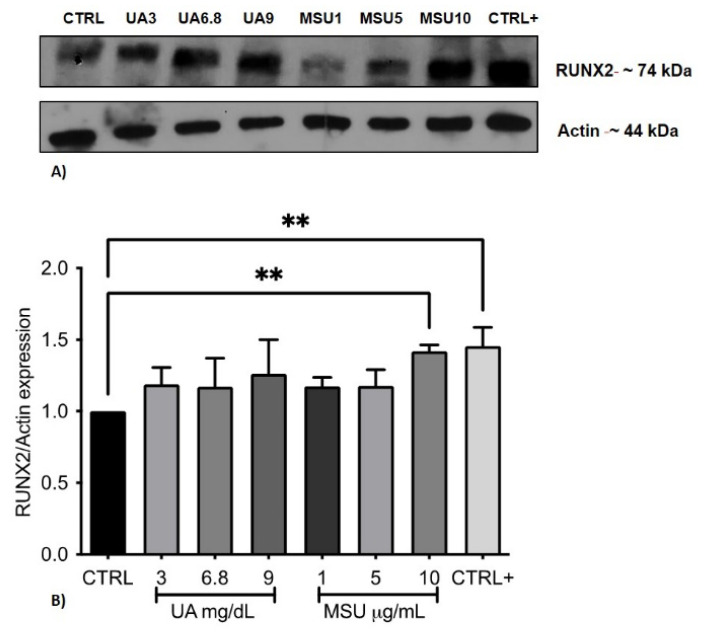
Effect of UA and MSU crystals on Runx2 expression. (**A**) Blot representative of the levels of Runx2 protein expression in relation to actin (internal control). (**B**) Graph of the densitometric analysis of Runx2 expression in the control group and treatment groups. The values in the columns are the average ± SD of at least 3 independent experiments; Dunnett’s test, ** p < 0.01.

The expression of Runx2 in cells treated with 3 and 6.8 mg/dL of sUA increased by 20% with respect to the control, and in cells treated with 9 mg/dL UA, expression increased by 30%; however, the difference was not significant. In cells stimulated with 9 mg/dL, an upward trend in Runx2 expression (8.3%) was detected with respect to cells stimulated with 3 and 6.8 mg/dL sUA. Treatment with 10 μg/mL MSU crystals resulted in a significant increase of 40% with respect to the control, which was similar to that in cells stimulated with the osteogenic medium. Treatment with 1 and 5 μg/mL MSU crystals resulted in an upward trend of 20% (Figure 5).

## 4. Discussion

In diarthrodial joints, the SM acts as a semipermeable membrane, controlling molecular traffic into and out of the joint space and maintaining the composition of synovial fluid. Besides FLSs and macrophage-like synoviocytes, the SM contains a subpopulation of MSCs [20].

SM can provide a heterogeneous source of MSCs for experimental studies. According to the definition provided by the International Society of Cell Therapy, the cells that we isolated from SM are considered MSCs because of their ability to adhere to plastic, their expansion when cultured in vitro, their ability to form colonies, and their fibroblast-like morphology [13].

The immunophenotypes of the SM-derived cells were CD90+, CD105+, and CD73+, and CD34-, CD14-, and CD4-. CD90 is highly expressed in cells derived from SM, indicating stem cells with multilineage capacity. The immunophenotype corresponds to those reported by Huang et al. [21], Sakaguchi et al. [22], Segawa et al. [23], Prado et al. [24], and Hatakeyama et al. [25], which indicates that the MSCs are characterized by positive expression of CD90, CD105, CD73, and CD44 (specific for stem cells) and negative expression of markers associated with the hematopoietic cell lineages CD45, CD34, CD11, CD14, and CD117, among the most common ones.

Immunomagnetic separation was used to enrich CD90+ cells. This tool provides an adequate method to obtain a satisfactory number of cells from a small part of tissue in a short time, allowing us to obtain homogeneous cultures of MSCs that expressed CD90 and CD105. These results are consistent with those of Jia et al. [26], who performed isolation and culture of MSCs derived from synovial fluid, and successfully purified them by MACS using the MSC surface marker CD90. Indeed, MACS is a useful technique for the purification of MSCs from synovial fluid or SM.

In this study, sUA and MSU crystals did not affect the viability of SM-MSCs at any of the doses for the prolonged times of cell stimulation. The sUA doses were used to simulate states of hypouricemia, normouricemia, and hyperuricemia, and the micro-doses of the MSU crystals were for long-term stimulation. In a previous report, using FLSs to evaluate the dose–response curve of MSU crystals from 60 to 100 ug/mL during 24 h, a non-significant decrease was observed with the lower dose [27]. For the current study, we proposed minimal doses of MSU crystals in order to assure SM-MSC survival and identify the long-term effects in the model.

Regarding inflammation, IL-1β was undetectable; nevertheless, IL-1β plays a crucial role in driving the transition from the acute phase of arthritis to the irreversible chronic phase [28]. Only low levels of IL-6 were quantified with respect to the control; therefore, our system did not induce inflammation at those doses. Evidence by Zheng et al. [28] shows that exposure of FLSs to MSU crystals (1 and 10 ug/mL) transiently induced a significant increase in IL-1β expression in a culture medium, with a peak at 6 h. Changes in IL-6 and TNF-α expression were not observed. Likewise, Braga et al. [29] incubated bone marrow-derived macrophages in the presence of sUA alone or with LPS (sUA+LPS) for 6, 24, and 72 h. The sUA+LPS induced IL-1β mRNA expression when compared to non-stimulated cells and cells stimulated with sUA alone. IL-1β expression was higher at 6 h and decreased with time.

Our findings confirm that low levels of inflammation could be attributed to the micro-doses of MSU crystals in the model, and the sUA needs an adjuvant to cause inflammation. On the other hand, some authors attribute the self-limiting phases of inflammation in gout to regulators such as miR-146a, because MSU crystals can be present within clinically noninflamed joints and extra-articular tissues in people with previous acute outbreaks and in tophaceous gout, suggesting that there are additional self-limiting regulatory mechanisms of inflammation [30].

Regarding the effect of UA and MSU crystals on mineralization, the alizarin red technique is quite sensitive and does not strongly reflect calcium deposits; however, Runx2 expression was significantly increased in cells treated with 10 µg/mL of MSU crystals; i.e., similar to that observed in cells in the osteogenic medium. Runx2 is a relevant biomarker, because medications such as allopurinol reduce osteoblast apoptosis, increase their viability, and reduce the risk of vascular calcification by decreasing Runx2 in animal models of hyperuricemia [31,32]. In a recent study by Naot et al. [33], factors such as PGE2 and TNF-α, secreted by macrophages stimulated with MSU crystals, reduced the viability of osteoblasts in a dose-dependent manner in long-term cultures (13 days); however, the doses ranged from 100 to 500 μg/mL, which decreased the expression of Runx2, suggesting that bone erosion is a result of direct and indirect effects, such as the stimulation of exosomes derived from neutrophils by MSU crystals, which has a negative effect on the viability of osteoblasts [34].

Therefore, our findings indicate that MSU crystals induce signals that could favor the osteodifferentiation of SM-MSCs in the long term. Our model reflects the late events that occur due to the signaling between crystals and SM-MSCs. The use of SM-MSCs in this study is novel; this is one of the first studies to use this cell type to assess the long-term effects of both sUA and MSU crystals, in order to identify their effects on the induction of bone. However, we identified some limitations that must be addressed in future research. It will be necessary to evaluate other markers of osteogenic activity, such as alkaline phosphatase and bone morphogenetic proteins, among others. If possible, increasing the doses of MSU crystals would allow an approximation of the severe conditions that occur in chronic gout.

## 5. Conclusions

MSU crystals in SM-MSCs modulated osteogenic differentiation through an increase in Runx2 expression; however, more studies are needed to corroborate these findings, in which other osteodifferentiation markers should be considered to contribute to the knowledge regarding the role of crystals in the chronic and erosive stages of gout.

## Supplementary Materials

The following supporting information can be downloaded at: https://www.mdpi.com/article/10.3390/medicina58121724/s1, Figure S1, Figure S2 and Figure S3.
