# Supplementary figures and images for: Osteogenic Potential of Monosodium Urate Crystals in Synovial Mesenchymal Stem Cells
Source: Medicina (Kaunas). 2022 Nov 24;58(12):1724. doi: 10.3390/medicina58121724 (PMC9786019; doi:10.3390/medicina58121724)

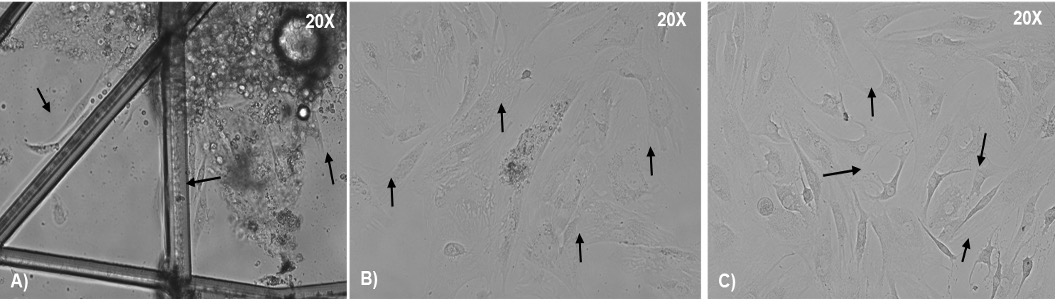

Supplement: Supplementary file 1 [file medicina-58-01724-s001.zip › FigS1.jpg]

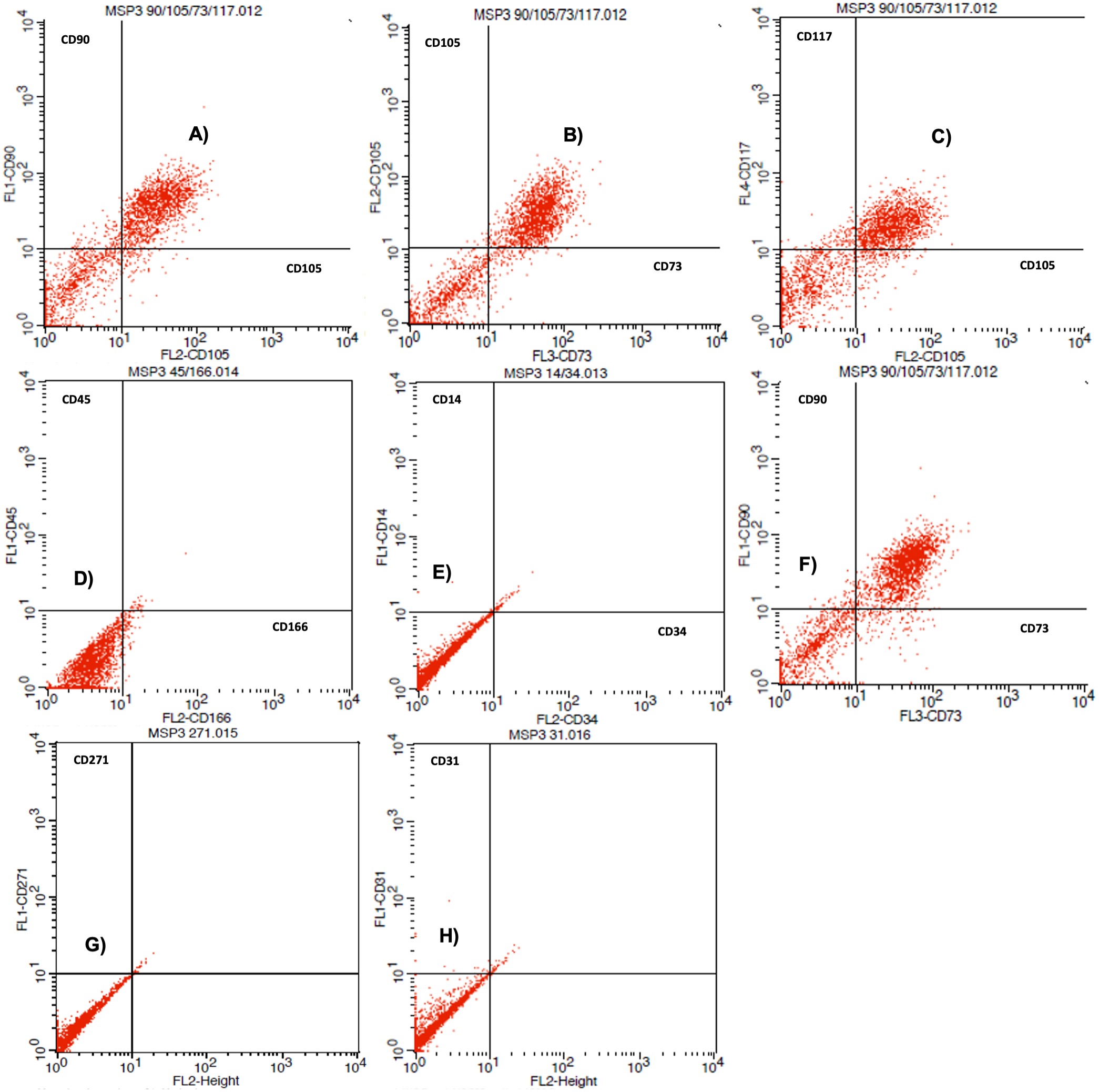

Supplement: Supplementary file 1 [file medicina-58-01724-s001.zip › FigS2.jpg]

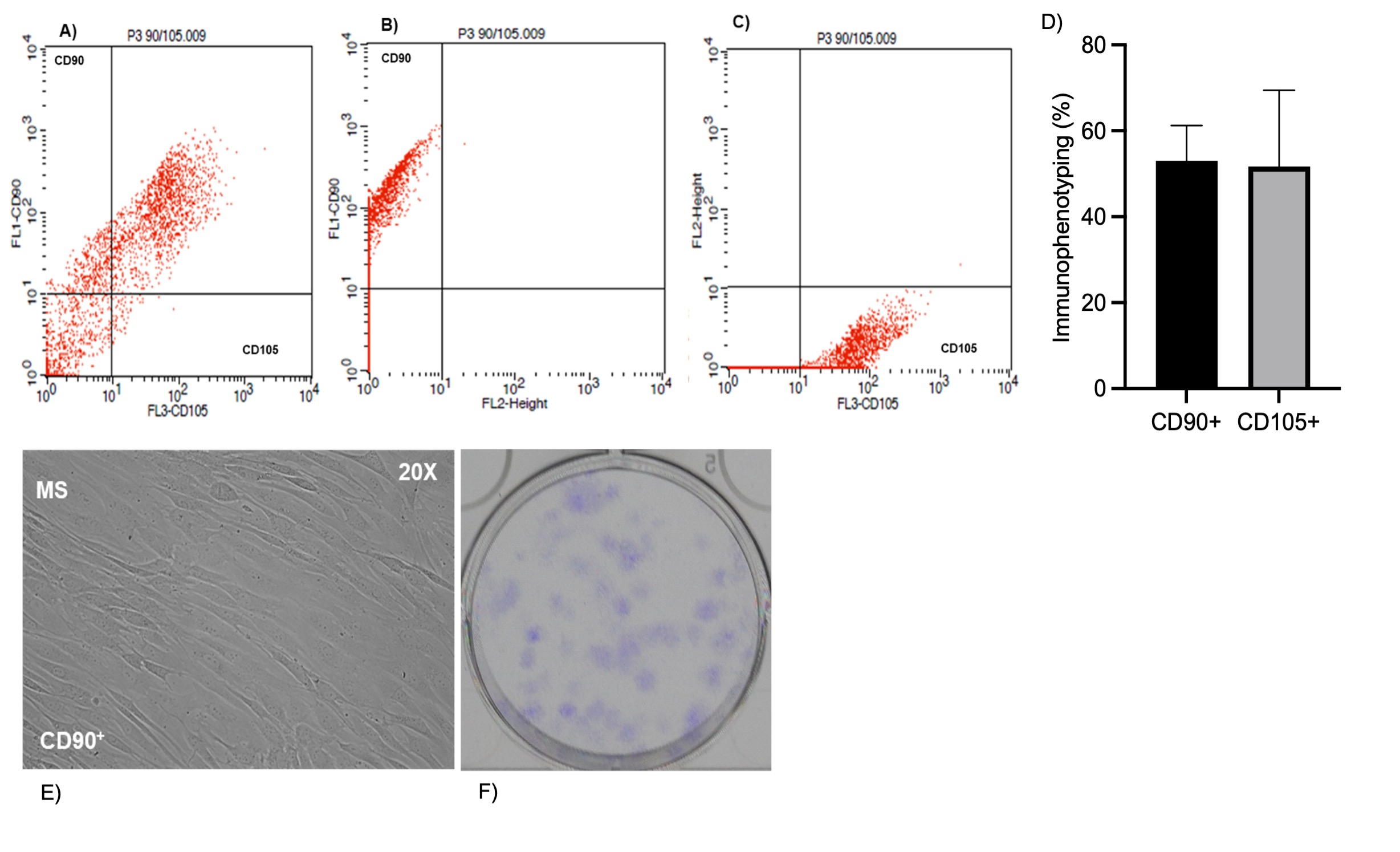

Supplement: Supplementary file 1 [file medicina-58-01724-s001.zip › FigS3.jpg]
